# WEEE Treatment in Developing Countries: Environmental Pollution and Health Consequences—An Overview

**DOI:** 10.3390/ijerph16091595

**Published:** 2019-05-07

**Authors:** Mentore Vaccari, Giovanni Vinti, Alessandra Cesaro, Vincenzo Belgiorno, Stefan Salhofer, Maria Isabel Dias, Aleksander Jandric

**Affiliations:** 1 Department of Civil, Environmental, Architectural Engineering and Mathematics, University of Brescia, 25123 Brescia, Italy; g.vinti001@unibs.it; 2 Sanitary Environmental Engineering Division (SEED), Department of Civil Engineering, University of Salerno, via Giovanni Paolo II, 84084 Fisciano (SA), Italy; acesaro@unisa.it (A.C.); v.belgiorno@unisa.it (V.B.); 3 Waste Management Institute, BOKU University, 1190 Vienna, Austria; stefan.salhofer@boku.ac.at (S.S.); aleksander.jandric@boku.ac.at (A.J.); 4 Centro de Ciências e Tecnologias Nucleares—C2TN, Campus Tecnológico e Nuclear, Polo de Loures, Instituto Superior Técnico, Estrada Nacional 10, km 139.7, 2696-066 Loures, Portugal; isadias@ctn.tecnico.ulisboa.pt

**Keywords:** environmental pollution, enrichment factor, e-waste, health impact, heavy metals, informal treatment, metalloids, organic pollutants

## Abstract

In the last few decades, the rapid technological evolution has led to a growing generation of waste electrical and electronic equipment (WEEE). Not rarely, it has been exported from industrialized to developing countries, where it represents a secondary source of valuable materials such as gold, copper, and silver. The recycling of WEEE is often carried out without any environmental and health protection. This paper reviews recent literature dealing with the informal treatment of WEEE in developing regions, gathering and analyzing data on concentration of both inorganic and organic pollutants in the environment. Open burning practices are revealed as most polluting ‘technology’, followed by mechanical treatment and leaching. Significant levels of pollutants have been detected in human bodies, both children and adults, working in or living in areas with informal WEEE treatment.

## 1. Introduction

In the recent decades, technology has largely contributed to the improvement of the quality of life, providing several benefits and opportunities in a variety of areas. Nevertheless, its rapid evolution has led to the fast disposal of a number of electric and electronic devices, referred to as waste electrical and electronic equipment (WEEE). 

WEEE stands as the residual stream characterized by the highest annual generation rate [1]. In 2016, the global production of WEEE was 44.7 million tons (Mt) and it is expected to exceed 50 million tons in 2021; however, only one-third of the WEEE generated is documented to be properly collected [2] and destined to recycling, in accordance with the legislative framework disciplining waste management in most high income countries.

Asia is the greatest producer of WEEE (18.2 Mt), followed by Europe (12.3 Mt), the Americas (11.3 Mt), Africa (2.2 Mt), and Oceania (0.7 Mt). Notwithstanding, the smallest overall generation, Oceania is reported as the region with the greatest specific waste generation (17.3 kg/inh. (inhabitant)/year), of which only 6% is documented to be properly collected and recycled. Europe is the second largest WEEE producer per inhabitant with an average of 16.6 kg/inh./year, but the collection rate reached the 35% in 2016. The Americas and Asia have comparable collection rates, but the annual specific WEEE production in Asia is 4.2 kg/inh., approximately half that recorded for the Americas. Africa generates only 1.9 kg/inh./year, but scattered information is available about the collection of the WEEE produced [2].

The collection of WEEE is a crucial step in providing its management in compliance with the waste hierarchy, pursuing material recycling as the preferred option for waste streams. 

The WEEE recycling chain usually consists of a mechanical pre-treatment step, aiming at the separation of the hazardous components from the valuable ones, which are further sent for refining via metallurgical techniques [3,4,5] and other recycling processes. In high-income regions, all the operations for WEEE recycling are formally identified, so that the intrinsic toxicity potential of some components [6] does not threaten both the environment and human health.

However, there is a large amount of WEEE generated globally, whose fate is unknown [2]. This portion of WEEE is likely to enter the so-called informal sector. In this context, the collection of WEEE and its separation as well as the extraction of valuable metals—like copper, gold, and silver—are carried out without any environmental and health protection against the potential burdens related to the hazardous substances contained in WEEE [7]. During the informal treatment, these substances may either be released into the environment or generate toxic emissions, posing severe threats for both the environment and human health [8,9]. 

Several studies report the contamination of soil, air, and water by heavy metals and organic contaminants in and around the informal working areas. The contamination of both water and sediments is documented in rivers close to informal WEEE recycling area such as Guiyu, in China [10]. Concentration of polycyclic aromatic hydrocarbons (PAHs) as high as 1231 mg/kg were detected in the soil of Wenling, an emerging e-waste recycling city in China [11]. In Indian workshop dusts, concentrations of lead were reported to range between 2360 and 10,900 mg/kg: such levels were approximately 5–20 times higher than the background ones [7]. The presence of these pollutants into the environment entails the potential risk for the human health of the exposed population. Both residents and workers may intake such contaminants either directly or indirectly. Yu et al. [12] recently pointed out the role of the informal recycling of WEEE on the release of heavy metals into the environment. In their work, authors considered the area of a former workshop in south China and compared the concentration of heavy metals in dust, soil, vegetable, and rice samples with the values detected when informal recycling of WEEE was going on. They found that the levels of heavy metals in the dust samples were lower than those reported when the WEEE was informally treated in that area; however, the concentrations of the same pollutants in soil, vegetables, and rice were still high. This condition made the exposure via crop consumption a relevant contributor to health risk in this area. Zeng et al. [13] extensively described the health effects by heavy metals in an e-waste recycling area, pointing out that major injury to diverse organism as well as genetic damage can occur in children.

The informal sector is mainly based in developing regions, where formal recycling is unlikely feasible due to the excessive costs related to its implementation [14], and it is largely fed by the WEEE produced in high-income countries. In most cases, sending WEEE to developed regions complies with the need to fulfil the implementation of the extended producer responsibility (EPR) while reducing the associated economic burdens. This kind of approach is also encouraged by the loopholes in current e-waste global regulations: the illegal e-waste flows from developed to developing countries are allowed under the guise of “donation” or “refurbishing” purposes [10]. Nevertheless, the demand for imported WEEE in developing countries has considerably increased due to the potential economic gain from the unregulated recycling [10]. 

Breivik et al. [15] highlighted that the 23% of WEEE generated in developed countries is exported to seven preferred developing countries. The 75–80% of the entire amount of the WEEE globally produced is transferred to the Asian and African developing regions [16], standing as the preferred destinations for the illegal traffic of WEEE, whereas the main source countries have been identified in Europe [17]. In China, due to the introduction of tighter legislation, the management of WEEE has gradually improved over time [18], while Ghana, Nigeria, South Africa, Vietnam, India, and the Philippines became attractive destination countries [17].

The geography of WEEE global movements is, thus, particularly complex and continuously changing, according to the different provisions and regulations that are entering into force, shifting the destination countries time to time. 

In the informal sector, the processing chain of WEEE still pursues the recovery of valuable components, but mechanical processing in the formal treatment is replaced by manual disassembly, whereas the refining stage occurs via either open burning or uncontrolled acid leaching. Poor process monitoring, as well as the absence of personal protective gear, accounts for both severe contamination of the environment where these practices take place and for the risk to human health of workers and people living in the surroundings of the informal working sites [19,20,21]. 

The informal sector involves more than 60% of the population employed worldwide. About 93% of them live in emerging and developing countries. For example, including agriculture, in Africa 85.8% of employment is informal, while in Europe less than 30% [22]. Focusing on the WEEE industry, over 700,000 people were employed in 2007 in China, 98% being in the informal recycling sector [23]; furthermore, more than 95% of recycled WEEE goes through the informal sector in India [24,25].

This work reviews recent literature dealing with the informal treatment of WEEE in developing regions, showing concentration of both inorganic and organic pollutants in the environment. Studies about contaminant presence in humans working in or living by WEEE treatment facilities and associated potential health effects are reviewed, as well.

## 2. Materials and Methods 

A systematic survey of the published literature was performed to look for the concentration of contaminants in the environment due to the informal management of WEEE in developing regions worldwide. 

Relevant search engines, i.e., ScienceDirect and Scopus, and a combination of search terms (i.e., WEEE, e-waste, developing countries, informal treatment) were used. Papers published since 2005 were examined to ensure the currency of the information. 128 papers were identified as potentially useful and initially analyzed (listed in the available Supplementary Material). Papers not regarding informal WEEE activities or not including concentrations of contaminants in the environmental media (i.e., soil, dust, sediment, air, water) were then excluded.

The searched inorganic pollutants were: Al, As, Ba, Cd, Co, Cr, Cu, Hg, Li, Mn, Mo, Ni, Pb, Sb, and Zn. The searched organic pollutants were: polybrominated diphenyl ethers (PBDEs), polychlorinated dibenzodioxins (PCDDs), polychlorinated dibenzofurans (PCDFs), polychlorinated biphenyls (PCBs), dioxin-like PCBs, and organophosphate flame retardants (PFRs). Both the inorganic and organic pollutants were chosen since usually mentioned as typical environmental pollutants in WEEE treatment activities and for their impacts on human heath [26,27,28,29]. Aluminum was chosen to calculate the enrichment factor discussed below [30].

Afterwards, 50 papers including data concerning 107 sites from all over the world were selected (Table 1). Six papers were review papers. In this paper, data from those reviews were integrated with the further studies to have a global vision.

In few cases (Table 1, sites with codes CG, CH, CN, DA, DB), data were taken into consideration although the scientific paper provided them as an average value from two or three locations. Indeed, in those cases, the values were considered anyway useful because the sites were from the same country and they had the same classification of WEEE treatment process.

The data were arranged according to the media sampled and analyzed in the literature sources. Soil, dust samples, sediments, air, and water samples. From the wider range of elements, four heavy metals—i.e., Cu, Ni, Pb and Zn—were selected for their stable chemical nature and because data for comparison are available. 

Some values represent single values, while others base on a larger number of samples. In the evaluation, all datasets (data from papers) were treated equally, as long as the sampling and background information could be identified. 

In order to evaluate differences in impacts between treatment technologies, each dataset was assigned to one of the categories (using the information given in the papers, partly scarce):

- Mechanical treatment: fragmentation, sieving, sorting, etc.
- Open burning: burning of compound materials with plastic content to release the metal content, e.g., cable burning
- Leaching processes: acid leaching, amalgamation
- Mixed: more than one treatment technology applied
- NA (not available): unknown, not explained in the paper

The data of Table 1 are sorted by countries and, then, by treatment process. In the last column there is a specific code for each site; in this way, the values showed in the Results section (Table 2, Table 3, Table 4, Table 5, Table 6, Table 7, Table 8, Table 9 and Table 10) are associated to those codes.

One of the more traditional approaches to evaluate the sources of trace elements is the analysis of elemental concentration ratios, particularly by comparing the ratios in the samples with those in a likely source material by calculating enrichment factors (EFs). EFs are widely used to estimate the anthropogenic impact on soil, and they are based on the normalization of analytical data against a reference element of which the occurrence in the environment has low variability, and are calculated as

EF(C) = (C/C_ref_)_sample_/(C/C_ref_)_UCC_ (1)

where *C* is the concentration of the studied chemical element and *C_ref_* is the concentration of the chemical element adopted as reference element (in this work Al). The reference element should not be influenced by anthropogenic activities and weathering processes [30], and one of the most commonly used is Al. The EFs relative to upper continental crust (UCC) values [73] have been used to understand the relative dominance of anthropogenic sources for any element (*C*) in soils, and in aerosols [74]. In general, EF~1 indicates the crust as primarily source, EF <1 indicates depletion, EF >1 indicates enrichment of the element considered thus pointing to multiple sources; EF >5 indicates dominance of anthropogenic sources. The higher the EF value, the more severe the anthropogenic contribution is. Studied samples can then be given a contamination category based on the enrichment factor.

## 3. Results

This section reports the concentration of pollutants measured in the sites where informal treatment of WEEE is carried out. When the numerical values had decimal places, they were reported with the first one, with the exception of the cases in which it would have excessively reduced the accuracy of the values (as in Table 6). Furthermore, when the last decimal place was the number 5, since several methods to rounding exist [75], it was decided to round half down when the previous was an even number and round up when the previous was an odd number.

Table 2 illustrates the concentration of heavy metals and metalloids in soils. It can be noted the high number of available data from 42 different sites. It can be seen how the majority of the data are related to Cd (from 39 sites), Cu (41 sites), Pb (39 sites), and Zn (39 sites). It can be noted that one site (code DQ) has the highest values for many heavy metals: the informal e-waste recycling area (with open burning activities) of Agbogbloshie, in Accra (Ghana) [67], in particular for As, Hg, Pb, and Zn.

Table 3 shows the concentration of heavy metals and metalloids in dust samples. A lower number of sites (22) with that datum in available in the literature. In this case, the majority of data are associated to the following pollutants: Cd (from 19 sites), Cu (19 sites), and Zn (18 sites). Furthermore, the highest pollution level comes from the e-waste recycling area of Zarfarabad in New Delhi (India) (code CT [26]) in particular for Cr, Cu, and Hg.

Table 4 shows the concentration of heavy metals and metalloids in sediments. In has to be highlighted the majority of the sediments are from rivers, with three exceptions (pond: codes AX, AY; Not Specified: code DJ). The available data comes from 12 sites. The majority of the data are related to the following pollutants: Cd (from 10 sites), Cu (10 sites), and Pb (12 sites). It can be noted that one site (code BT) has the highest values for many heavy metals: the Taizhou informal e-waste recycling site in China [31], in particular for As, Cd, Cr, and Cu. 

Table 5 illustrates the concentration of heavy metals and metalloids in air samples. In this case, the data refer to only six sites. The majority of the data are related to the following pollutants: Cd (from 6 sites) and Pb (6 sites). It can be noted that one site (code AA) has the highest values for many heavy metals: the printed circuit board recycling workshop of Guiyu (China) [31,32]. 

Table 6 shows the concentration of heavy metals and metalloids in water samples. The data come just from four sites. A good amount of data is related only to Pb (from four sites). The number of sites is too low for any comparative consideration.

Table 7 illustrates the concentration of organic pollutants in soils. In this case the number of available data come from 16 sites. In this case, it was not possible to define a site as ‘more polluted than others’, in particular due the low number of different pollutants.

Table 8 shows the concentration of organic pollutants in dust. The data come from 13 sites; the majority of the data is related to PCB (from 12 sites), which reaches 34 μg/g as a maximum value.

Table 9 shows the concentration of organic pollutants in river sediments. The available data are from only three sites and refer to PBDEs and PCDD/Fs.

Table 10 illustrates the concentration of organic pollutants in air samples. The available data come from seven sites. The majority of the data regards PCDD/Fs (from four sites).

The concentration of organic pollutants in the water is not shown because of the very few and non-significant available data. 

## 4. Discussion

**Figure 1:**
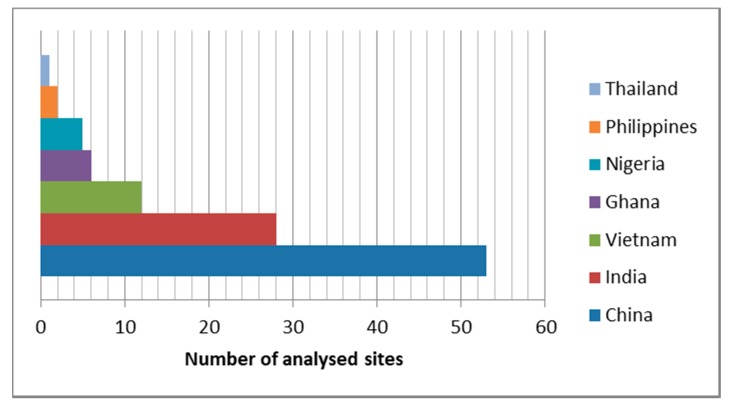
Number of sites analyzed in the selected countries.

As shown in Figure 1, most of the sites analyzed are located in China (49.5%) and India (26.2%). The others were from Vietnam (11.2 %), Ghana (5.6%), Nigeria (4.7%), Philippines (1.9%), and Thailand (0.9%). The fact the majority of the studies and data origin from China and India can be explained taking into account that, on one hand the two countries are the most populated in the world, each of them with more than 1 billion people, on the other hand they are no longer poor countries and local research centers carry out advanced studies also in this field. Moreover, in China and India, underdeveloped areas remain where informal treatment of WEEE is a widespread economic activity. 

**Figure 2:**
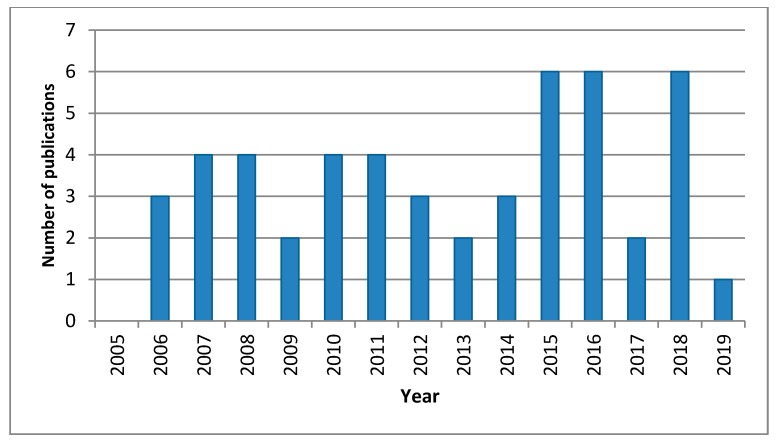
Number of annual publications, from 2005 to 2019.

Figure 2 shows the number of annual papers included in Table 1, regarding both informal WEEE activities and concentrations of contaminants in the environmental media, from 2005 to 2019. As it can be seen, excluding 2005 and 2019, the papers published per year are always between 2 and 6.

Metals are natural constituents of the crust of the Earth and may be present in varying concentrations in different ecosystems [76]. Indeed, in rather pristine areas, the main source of metals in soils is the weathering of the geological substrate via pedogenesis processes [77], consequently in these areas their concentration has a site-specific nature. However, human activities have drastically changed the biogeochemical cycles and balance of some heavy metals, mainly locally.

Considering rather pristine areas, for instance the average concentration of heavy metals in the atmospheric air over the Arctic seas, in particular the White Sea, was less than 0.1 ng/m^3^ for Cd, less than 1 ng/m^3^ for Cr and Pb, less than 10 ng/m^3^ for Cu and Zn [78]. Of course, it has to be considered sometimes in apparently uncontaminated areas it can be the influence of long-range atmospheric transport of metals [56].

Regarding the organic pollutants, in particular POPs (persistent organic pollutants) as polychlorinated dibenzo-p-dioxins and dibenzofurans (PCDDs/PCDFs) and dioxin-like PCBs (dl-PCBs) in uncontaminated areas such as the Pacific Islands, it was found a concentration in air lower than 0.1 pg/m^3^ in terms of TEQ [79].

Soils may become contaminated due to the accumulation of heavy metals [80], particularly those resulting from the disposal/treatment of metal wastes and especially due to the use of wastewater, which leads to changes in some soil physicochemical characteristics and heavy metal uptake by food crops [81]. In addition, atmospheric deposition and an increase in the soil organic carbons due to industrial activities may affect the availability of heavy metals. Previous studies [82,83,84] demonstrate the health problems related with consumption of plants grown on wastewater-irrigated soils contaminated with heavy metals, particularly in China and India. A thorough evaluation of soils and dust contamination is considered as crucial for human health risk assessment [27] enabling a better comprehension of exposure parameters.

Informal treatment of WEEE can include different processes and technologies. Mechanical treatment is the most widely used recycling technology and encompasses a variety of activities ranging from manual sorting, disassembly for re-use, dismantling, shredding, sieving, screening, and similar processes. The scope and organization of mechanical treatment depends highly on local circumstances—i.e., quantity of available electronic waste—the price of manual labor (hourly wages), market prices for secondary raw materials, available transportation (land or sea), and similar (c.f. [14,85]). The informal mechanical treatment often coexists with open burning practices. In general, the less efficient mechanical treatment is in place, the more likely and wider in scope open burning practices are.

The practice of open burning of waste electronics is a crude technological quick fix mainly carried out for two purposes: either to remove plastics and isolate metals—e.g., burning of wires, plastic metal assemblies, PCBs, etc.—or in order to reduce the volume of unwanted materials (c.f. [39,53,86]). The open burning causes brutal damage to environment, workers and local residents via inhalation, dermal exposure, and oral intake. Thus, high levels of toxic metals (e.g., lead, cadmium, mercury) and organic pollutants (e.g., polychlorinated biphenyls—PCBs; polybrominated dibenzo-p-dioxins/dibenzofurans—PCBB/Fs) can be found in air, water and sediments near recycling sites [87,88].

Leaching processes are part of the final treatment of e-waste and almost exclusively oriented towards copper and gold recovery. Since its efficiency depends on the specific surface of particles, several pre-treatment steps to reduce the plastics share—e.g., mechanical (size reduction, sieving, classification, etc.) and burning—are prerequisite for the leaching process. Input material for leaching processes comprises of various types of PCBs, gold connectors, and other electronic components with high copper and gold concentrations. 

For gold recovery from high-gold-contained parts, Keller [89] described the detailed processes and applied techniques at informal gold recovery facilities which consists: (1) “cyanide leaching” for low grade material and (2) “mercury amalgamation” for high grade material. Cyanide leaching consists of a leaching step where potassium or sodium cyanide is added into a container filled with hot water. Then, a gold separation step is takes place by using aluminum foils and silver salt. The last step is purification to obtain pure gold. 

**Figure 3:**
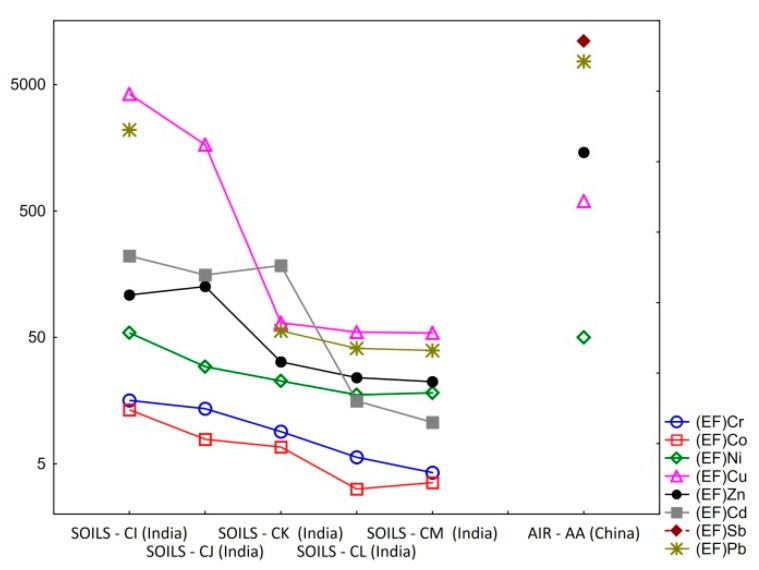
Enrichment factors for trace elements of soils and aerosols suggesting a huge contribution from anthropogenic sources.

Table 11 and Figure 3 show the EF for soil and air samples of the analyzed sites. The higher values were obtained for Cu, Pb, and Cd; in the cases of soils, and Sb, Pb, Zn, and Cu; in the case of air. Cr and Co have the lower enrichment factors (3–15), but are high, and, like all the other chemical elements studied, clearly point to a dominance of anthropogenic sources. Considering the enrichment factors categories proposed by Barbieri [90] soil/dust quality state can be indicate by different classes, ranging from EF < 2 (deficiency to minimal enrichment) to EF > 40 (extremely high enrichment). The data analyzed mostly point to an extremely high enrichment, or in the case of Cr, Co, Ni, and Cd from India samples a significant enrichment, allowing us to scrutinize the content of a given substance in the environment and to detect high anthropogenic influence.

Comparative analysis shows that all the studied elements are higher than the corresponding values of world common trace metal range in soils, aerosols, and water [91,92,93,94,95] creating severe adverse effects on ecosystems and human health.

This fact is particularly enhanced by the difference between the ‘background’ used and the upper threshold enrichment factors obtained for different heavy metals and soils and air.

Even the EFs calculated are a rough approximation due to the lack of data. In general, they enable the establishment of comparisons between the severity of impact between regions, and between treatment processes (when available), making it possible to clearly enhance the presence and intensity of anthropogenic contaminant deposition on surface soil and air.

**Figure 4:**
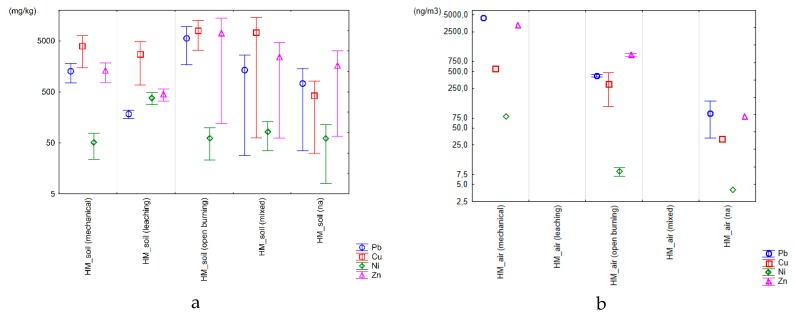

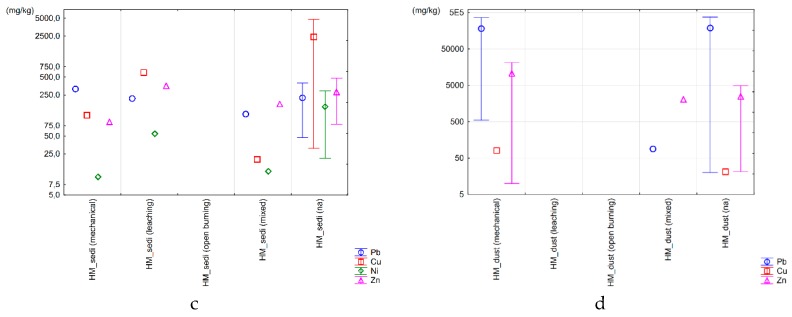
Range plot of heavy metal variables with the minimum, mid-point, and maximum values for the soils (**a**), air (**b**), sediment (**c**), and dust samples (**d**) according with the treatment processes (mechanical, leaching, open burning, mixed, na: not available).

In Figure 4, the range of heavy metals concentrations sorted by treatment processes and the type of samples are presented. In the case of soils samples, Cu has the higher range of values, followed by Pb, and Zn. Ni has the lower values and the lower range. The higher concentrations of heavy metals in soils have been found when using open burning and mixed technologies. The range of values are lower when mechanical treatment and leaching processes are applied. In the case of air samples, the mechanical treatment leads to higher concentrations of heavy metals, especially Zn and Pb. In the sediment samples, higher contents of Pb and Cu occur independently of the treatment procedure, with a tendency to become higher when bleaching procedure is used (most of the data we have do not mention the technological treatment). For dust and water samples, we do not have enough comparable data, but anyway higher range values were found for Pb and Zn, particularly when using mechanical treatment. 

Considering the cases for which there are more available data, it can be concluded that, in general, the open burning practices are the most polluting technology, followed by mechanical treatment and leaching.

The wide range of parameters as well as the different characteristics of the sites suggest that a site-specific analysis is necessary to evaluate the environmental and health impact of an informal WEEE treatment facility. Nevertheless, when putting those results in the larger picture, the risky nature of those processes and practices come clear. Compared to background data (here: world average soil data by Alloway [96]) the values from the samples in literature exceed background (average) value by far. All the technologies analyzed are heavy polluting practices, where open burning is the most polluting one.

In general, to assess the health risk resulting from exposure to a contaminant requires knowledge of both the dose that a person intakes as a result of exposure and the potential health effects of the contaminant [97].

Regarding the pollutants showed in the previous tables, many studies describe their effects on human health. For instance, Zahra et al. [98] took into consideration the toxic effects of heavy metals. Cu is required in trace amounts by the human body, but an excess of it can cause damage to cellular components. In particular, copper can be bio-accumulated and high concentrations of copper in liver, kidneys, brain, and cornea, leading to Wilson’s disease [98]. Lead is a toxic heavy metal, as well. Children with high lead exposure, typically at blood levels of 80 µg/dL or more, may present encephalopathy. A further relevant aspect of lead toxicity is peripheral neuropathy, which has been observed in house painters and other people with occupational exposure to lead [98]. Cadmium is a persistent pollutant causing kidneys and bones related diseases as a decrease in glomerular filtration rate, renal proximal tubule, increased rate of osteoporosis, high rate of fractures, low bone mineralization, and pain in bones known as Itai-Itai disease [98].

The health consequences of metals exposure to e-waste in China, in particular in Guiyu town, was described by Song and Li [99]. For instance, increments in negative health outcomes associated to pregnant women exposed to heavy metals from e-waste were observed in children and neonates. 

Considering the organic compounds, Quinete et al. [100] analyzed the potential health effects in humans related to polychlorinated biphenyl (PCB) metabolites in blood, highlighting that epidemiological and experimental studies reported that PCBs disturb TH homeostasis and the cerebral nervous system in humans and rodents. Further negative health effects of PCBs on animals and humans have been observed, including carcinogenicity.

A study conducted by Cogliano et al. [101] summarized the link between individual chemicals (as heavy metals and organic compounds) and specific cancer sites within the human body, taking as reference data from International Agency for Research on Cancer (IARC) monograph series. It is important to consider that, besides concentration of contaminants, to define a comprehensive conceptual model necessary to develop an accurate and complete health risk assessment, further site-specific information is needed; namely, the characteristics of the environmental matrix involved, the possible exposure, and the distance respect the point of exposure (POE). Indeed, a comprehensive model includes all four components of the risk calculation: release, transport, exposure, and consequence [97].

**Figure 5:**
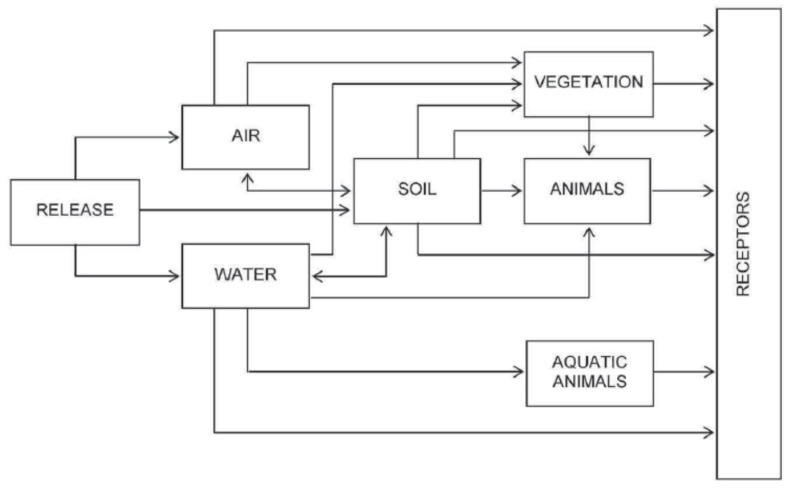
Generic environmental pathways and compartments (from [97]).

Typical environmental transport pathways are represented in Figure 5 in terms of discrete environmental compartments. 

Taking into consideration Figure 5, the contaminants released in the environment through informal WEEE activities may reach people living or working in the surrounding areas. Indeed, exposure refers to the contact of humans with contaminants. For environmental contaminants, typical exposure pathways are [97]: inhalation of contaminated air; ingestion of contaminated water, food, or soil; dermal contact with a contaminated medium such as water or soil.

It has to be considered that several studies showed as traces of the pollutants were present in the body of people working in or living by informal WEEE treatment areas. For instance, a study conducted in Ghana [102] analyzed the urine samples from 20 workers at the e-waste recycling site in Agbogbloshie (Accra). The study highlighted the presence of many heavy metals and metalloids in the urine. In particular, concentrations of Fe, Sb, and Pb in urine of e-waste recycling workers were significantly higher than those of reference sites without informal e-waste activities. 

A further study [103], conducted in Agbogbloshie as well, analyzed the level of dibenzo-p-dioxins and dibenzofurans in blood of informal e-waste recycling workers. The measured concentrations resulted significantly higher than those found in a control group of people of Accra living without direct exposure to e-waste dumps/recycling sites activities. 

A study conducted in China [104] revealed high serum levels of polybrominated diphenyl ethers in the blood of a study population of 23 workers who manually dismantled and ‘recycled’ e-waste such as personal computers and mobile phones on a daily basis. The median value was 382 ng/g lipid weight, significantly higher than the median value (158 ng/g) measured in 26 farmers in a village located 50 km away from the recycling site. 

Two studies [105,106], both conducted in Guiyu (China), where improper dismantling and combustion of e-waste are common, found elevated levels of lead in the blood of children (2–7 years of age). The level was significantly higher than in Haojiang, a reference site which lacks e-waste pollution. Similar results were obtained in a further study [53] concerning Pb and Cd.

Furthermore, a recent study [107] conducted in China compared concentration of heavy metals in human urine samples of people living in Qingyuan City, in e-waste dismantling areas, with the concentration in people from a rural reference area. The study found a geometric mean concentration significantly higher in the people from e-waste dismantling area for Cd (2.12 μg/L), Cu (22 μg/L), Pb (4.98 μg/L), and Sb (0.20 μg/L). 

In India, a study [108] determined residue levels of PBDEs in serum from e-waste recycling workers significantly higher than those in serum from residents living near the coastal area; concentrations of PCB and OH-PCB congeners in serum were higher as well, but not significantly.

The abovementioned studies demonstrate that contaminants released in the environment by informal WEEE treatment facilities may reach people living or working in the surrounding areas. In predictive studies, transport of pollutants in the environment and their attenuation during the pathways (e.g., for degradation, dispersion, diffusion) can be evaluated by different models [97,109,110]. In this way, the concentrations of contaminants at the point of exposure and, consequently, the health risk for humans can be foreseen. This approach, for instance, was used by Vaccari et al. [110] to assess potential health risks given by groundwater contamination of leachate originated in dumpsites in developing countries.

A health risk assessment of the workers (both adults and children) exposed to heavy metals and metalloids in e-waste recycling sites in India was performed by Singh et al. [27]. A toxic risk (>1) for children was estimated due to exposure to As, Cu, Cr, and Pb; whereas a carcinogenic risk >10^−5^ was determined for adults due to exposure to As, Cd, Cr, Ni, and Pb.

Recently, Yu et al. [12] evaluated the health risk given by a former e-waste recycling area in China. For children, the non-carcinogenic risk was higher than 1 for Cd, Cu, and Pb, while the carcinogenic risk (value >10^−5^) was found for Pb. For adults, the non-carcinogenic risk was higher than 1 for Cd and Pb, while the carcinogenic risk was determined for Pb.

## 5. Conclusions

In the last few decades, the rapid technological evolution led to a growing production of WEEE. Not rarely it has been exported from industrialized to developing regions [15], where the informal sector is mainly based [14,22]. The related WEEE treatment activities are usually carried out under poorly controlled conditions resulting in severe environmental pollution [20]. Through this paper, data were collected in the areas in which WEEE informal activities are conducted, in terms both of inorganic and organic compounds in the environment; in particular in air, dust, soil, water, and sediment. In order to evaluate the differences in impacts between the treatment technologies applied, each dataset was assigned to one of the categories, using the information given in the papers. The categories were: mechanical treatment, open burning, leaching processes, mixed (where more than one technology was applied), and not available (where not enough information was available to assign the data to one of the categories). The data originate from seven countries, with a majority for China and India. Most data were available for heavy metals and metalloids in soils. The results of this review show that open burning practices are the most polluting ‘technology’, followed by mechanical treatment and leaching. 

Using the enrichment factor for soil and air samples, the comparative analysis shows that all elements considered are in a higher range than the corresponding values of world common trace metals. Finally, it was highlighted how the spread of pollutants in informal WEEE treatment facilities affects human health through transportation phenomena and exposure pathways. Indeed, several studies have highlighted high levels of both organic and inorganic contaminants in the bodies of people working in or living by those areas. Since the presence of those contaminants in humans may cause several toxic and carcinogenic diseases, urgent measures should be adopted to improve WEEE treatment in developing countries and reduce its environmental and health impacts.

## Figures and Tables

**Table 1:** Location, site description, treatment process, reference information and code chosen related to informal e-waste treatment in developing countries and areas in the world.

Location	Site Description	Treatment Process	Reference	Code
Guiyu (China)	Printed circuit board recycling workshop	Mechanical treatment	[31,32]	AA
Guiyu (China)	Family-run e-waste recycling workshops	Mechanical treatment	[31,33]	AB
Guiyu (China)	Family-run workshops in e-waste-recycling impacted area	Mechanical treatment	[31,34]	AC
Guiyu (China)	E-waste recycling sites (from Lianjiang River-1)	Mechanical treatment	[31,35]	AD
Wenling in Taizhou (China)	Paddy soil in an e-waste recycling area	Mechanical treatment	[31]	AE
Longtang (China)	E-waste disassembling workshops	Mechanical treatment	[31]	AF
Shijiao (China)	E-waste disassembling workshops	Mechanical treatment	[31]	AG
Qingyuan (China)	E-waste recycling area	Mechanical treatment	[28,36]	AH
Guiyu (China)	Street characterized by residential and commercial buildings involved in e-waste recycling	Open burning	[37,38]	AI
Guiyu (China)	Street characterized by residential and commercial buildings involved in e-waste recycling	Open burning	[39,40]	AJ
Guiyu (China)	E-waste recycling areas influenced by dumping–burning	Open burning	[39]	AK
Longtang (China)	E-waste open burning sites	Open burning	[31]	AL
Longtang (China)	E-waste incineration site	Open burning	[31]	AM
Taizhou (China)	E-waste recycling area	Open burning	[19,41]	AN
Qingyuan (China)	E-waste open incineration site	Open burning	[42]	AO
Guiyu (China)	Acid leaching of printed circuit boards site	Leaching processes	[37]	AP
Guiyu (China)	E-waste acid leaching treatment area	Leaching processes	[28,43]	AQ
Guiyu (China)	E-waste acid leaching treatment area	Leaching processes	[43,44]	AR
Guiyu (China)	Abandoned e-waste workshop	Leaching processes	[45]	AS
Guiyu (China)	E-waste recycling sites (from Lianjiang River-3)	Mixed	[31,35]	AT
Guiyu (China)	Lianjiang River, area related with circuit board, acid and burning processing of E-waste	Mixed	[39]	AU
Guiyu (China)	E-waste dumpsite soil	NA	[46]	AV
Guiyu (China)	Roadside soil	NA	[31,46]	AW
Guiyu (China)	E-waste recycling sites (from duck pond-A)	NA	[31,35]	AX
Guiyu (China)	E-waste recycling sites (from duck pond-B)	NA	[31,35]	AY
Guiyu (China)	E-waste recycling sites (from Lianjiang River-2)	NA	[31,35]	AZ
Guiyu (China)	E-waste recycling sites (from Lianjiang River)	NA	[31]	BA
Guiyu (China)	E-waste recycling sites (from Nanyang River)	NA	[31]	BB
Guiyu (China)	E-waste recycling area	NA	[28,47]	BC
Guiyu (China)	E-waste recycling area	NA	[28,48]	BD
Guiyu (China)	Circuit board recycling workshops	NA	[39,49]	BE
Guiyu (China)	Roads adjacent to E-waste workshops	NA	[39,49]	BF
Guiyu (China)	Lianjiang River, near e-waste recycling area	NA	[39,50]	BG
Guiyu (China)	Nanyang River, near E-waste recycling area	NA	[39,50]	BH
Guiyu (China)	Rivers near E-waste recycling area	NA	[39,51]	BI
Guiyu (China)	E-waste recycling site	NA	[19,52]	BJ
Guiyu (China)	Area near e-waste recycling workshop	NA	[45]	BK
Guiyu (China)	E-waste recycling area	NA	[53]	BL
Guiyu (China)	Area near informal E-waste workshops	NA	[54]	BM
Guiyu area (China)	Workshops and houses involved in E-waste recycling activities	NA	[55]	BN
Qingyuan (China)	Former e-waste recycling area	NA	[12]	BO
Qingyuan (China)	Area near informal E-waste workshops	NA	[54]	BP
Taizhou (China)	E-waste recycling area	NA	[31,56]	BQ
Taizhou (China)	E-waste recycling area	NA	[31,57]	BR
Taizhou (China)	Agricultural soils near e-waste recycling workshops	NA	[31,58]	BS
Taizhou (China)	E-waste recycling site (from Nanguan River)	NA	[31]	BT
Taizhou (China)	E-waste recycling site	NA	[39,59]	BU
Taizhou (China)	E-waste treatment indoor environment	NA	[19,60]	BV
Longtang area (China)	Workshops and houses involved in E-waste recycling activities	NA	[55]	BW
Longtang area (China)	Workshops and houses involved in E-waste recycling activities	NA	[55]	BX
Longtang area (China)	Workshops and houses involved in E-waste recycling activities	NA	[55]	BY
Wenling (China)	Simple household E-waste recycling workshops	NA	[11,44]	BZ
Dali area (China)	Workshops and houses involved in E-waste recycling activities	NA	[55]	CA
Chennai (India)	E-waste sites involved in dismantling and shredding of E-wastes	Mechanical treatment	[61]	CB
Zarfarabad in New Delhi (India)	E-waste recycling workshops with solder activities	Mechanical treatment	[26]	CC
Shashtri Park in New Delhi (India)	E-waste recycling workshops with solder activities	Mechanical treatment	[26]	CD
Brijgang in New Delhi (India)	Cathode-ray tube storage shed	Mechanical treatment	[26]	CE
Brijgang in New Delhi (India)	Open-air cathode-ray tube storage area	Mechanical treatment	[26]	CF
New Delhi, Mumbai and Chennai (India)	E-waste recycling sites characterized by dismantling activities	Mechanical treatment	[62]	CG
New Delhi, Mumbai and Chennai (India)	E-waste recycling sites characterized by shredding and grinding activities	Mechanical treatment	[62]	CH
Mandoli (India)	E-waste recycling site	Mixed	[63]	CI
Mandoli (India)	E-waste dumping site	Mixed	[63]	CJ
Mandoli (India)	An area 50 m away from e-waste recycling site	Mixed	[63]	CK
Mandoli (India)	An area 100 m away from e-waste recycling site	Mixed	[63]	CL
Mandoli (India)	An area 500 m away from e-waste recycling site	Mixed	[63]	CM
Chandigarh and Ludhiana (India)	E-waste dismantling workshop and general scrap dealer dismantling areas	Mixed	[27]	CN
Bangalore (India)	E-waste site in slum	NA	[64]	CO
Bangalore (India)	E-waste site	NA	[64]	CP
Bangalore (India)	E-waste site	NA	[64]	CQ
New Delhi (India)	E-waste battery recycling workshop	NA	[26]	CR
Shashtri Park in New Delhi (India)	Street near E-waste recycling workshop	NA	[26]	CS
Zarfarabad in New Delhi (India)	E-waste separation workshop	NA	[26]	CT
Shashtri Park in New Delhi (India)	E-waste recycling activities	NA	[26]	CU
Shashtri Park in New Delhi (India)	E-waste separation workshop	NA	[26]	CV
Kailash Nagar in New Delhi (India)	Street in an area without workshops	NA	[26]	CW
Safourjung in New Delhi (India)	Street in residential area	NA	[26]	CX
Buradi in New Delhi (India)	E-waste battery recycling workshop	NA	[26]	CY
Chennai (India)	E-waste sites with workshops engaged in metal recovery operations	NA	[61]	CZ
New Delhi, Mumbai and Chennai (India)	E-waste recycling sites for metal recovery	NA	[62]	DA
New Delhi, Mumbai and Chennai (India)	E-waste recycling sites	NA	[62]	DB
Gaziabad (India)	E-waste recycling areas with workshop	NA	[26]	DC
Bui Dau village (Vietnam)	E-waste processing area	Mechanical treatment	[44]	DD
Bui Dau village (Vietnam)	Open burning sites in an E-waste-processing area	Open burning	[65]	DE
Bui Dau village (Vietnam)	Open burning site in an E-waste recycling area	Open burning	[66]	DF
Bui Dau village (Vietnam)	Wires and cables open-burning area	Open burning	[29]	DG
Bui Dau village (Vietnam)	E-waste-processing workshop sites	NA	[65]	DH
Bui Dau village (Vietnam)	E-waste-processing area	NA	[65]	DI
Bui Dau village (Vietnam)	A river near an e-waste recycling workshop	NA	[65]	DJ
Bui Dau village (Vietnam)	E-waste recycling area	NA	[66]	DK
Bui Dau village (Vietnam)	E-waste recycling workshop	NA	[66]	DL
Bui Dau village (Vietnam)	A river near an e-waste recycling area	NA	[66]	DM
Bui Dau village (Vietnam)	E-waste processing site	NA	[29]	DN
Bui Dau village (Vietnam)	Areas adjacent to E-waste-processing workshops	NA	[29]	DO
Agbogbloshie, Accra (Ghana)	E-waste open burning area	Open burning	[8]	DP
Agbogbloshie, Accra (Ghana)	E-waste open burning area	Open burning	[67]	DQ
Agbogbloshie, Accra (Ghana)	E-waste recycling area, in correspondence of plumes emanating from the burning of e-wastes	Open burning	[68]	DR
Agbogbloshie, Accra (Ghana)	E-waste recycling area	NA	[68]	DS
Agbogbloshie, Accra (Ghana)	E-waste recycling area without burning activities	NA	[8]	DT
Agbogbloshie, Accra (Ghana)	E-waste recycling area without burning activities	NA	[67]	DU
Ojo (Nigeria)	E-waste dismantling area (during dry season)	Mechanical treatment	[69]	DV
Ojo (Nigeria)	E-waste dismantling area (during wet season)	Mechanical treatment	[69]	DW
Ojo (Nigeria)	E-waste recycling area (during dry season)	Open burning	[69]	DX
Ojo (Nigeria)	E-waste recycling area (during wet season)	Open burning	[69]	DY
Ojo (Nigeria)	E-waste dumpsite	NA	[26,70]	DZ
Manila (Philippines)	E-waste treatment area	NA	[44,71]	EA
Manila (Philippines)	E-waste recycling sites	NA	[71]	EB
Bangkok (Thailand)	Household workshops in which are dismantled E-waste	Mechanical treatment	[72]	EC

**Table 2:** Concentration of heavy metals and metalloids in soils (values expressed in mg/kg).

Code	Al	As	Ba	Cd	Co	Cr	Cu	Hg	Li	Mn	Ni	Pb	Sb	Zn
AE				0.3			41.1				39.9	48.3		137.0
AF				39.3			6371.5					1635.4		3039.6
AG				21.3			4000					943.7		2044.8
AH		80.2		6.3			2159.3	1.4			78.1		576.3	1366.0
AL				10.3		63.3	4850.6				100.3	1714.5		1016.7
AM				17.1			11,140					4500		3690
AO				17.1		68.9	11,140				60.1	4500		3690
AQ				1.4	11.9	7.4	684		22.4		278	223	1706	573
AR				1.4			684					222.8	1706	572.8
AS		26.0		1.2		2600	4800	0.2		300	480	150	1100	330
AV				32.0		153.6	787.7			374.1	114.2	1431		
AW				5.8		12.2	683.8			461	26.8	540.9		
BC		4.7		0.1	10.8	58.1	50.0	1.2	45.3		57.0	77.5	1.4	102
BD				1.3			25.8					71.6		99.3
BK		6.1		0.4		51	48	0.05		180	22	93	9.9	90
BO				2.4			97.0				8.1	53		104
BQ		4.1		1.2		6.1	98.8	0.3			34.6	55.8		
BR				6.4		26.8	256.4			366.56		46.8		209.8
BS				1.8		61.4	98.7				40.7	115.1		163.4
BZ				4.4			327					313		299
CI	8822.1	12.8		1.1	13.2	83.6	6734.9	0.1			1465	2134.0		416.3
CJ	14,142.6	17.1		1.3	12.4	115.5	4291.6	0.1			126.5	2645.3		776.8
CK	6476.4	3.8		0.7	4.9	34.8	77.0	nd			44.7	40.3		90.3
CL	6538.8	nd		0.1	2.3	22.0	65.3	nd			35.1	29.6		68.4
CM	6432.0	nd		0.04	2.6	16.4	63.4	nd			35.7	27.9		62.5
CN		40.0	976.4	8.3	19.4	287.2	145,434				130.2	1615.8		4737.7
CO					11	73	592			449				326
CP					14	54	429			619				192
DD				1			1520			509		759	46	761
DE		10 ^a^		0.3 ^a^	7.6 ^a^		340 ^a^		25 ^a^	530 ^a^	23 ^a^	90 ^a^		120 ^a^
DH		7.4 ^a^		0.4 ^a^	6.7 ^a^		130 ^a^		13 ^a^	300 ^a^	23 ^a^	89 ^a^		200 ^a^
DI		8.2 ^a^		<0.25 ^a^	6.4 ^a^		31 ^a^		18 ^a^	130 ^a^	17 ^a^	35 ^a^		66 ^a^
DQ		177.2 ^b^	218.2 ^b^	11.5 ^b^	26 ^b^	9.1 ^b^	12,450 ^b^	115 ^b^		102.5 ^b^		9475 ^b^	279.2 ^b^	14,025 ^b^
DU		43.8 ^b^	48.2 ^b^	2 ^b^	80.15 ^b^	3.1 ^b^	766.3 ^b^			81.7 ^b^		533.3 ^b^	8 ^b^	3205 ^b^
DV				10.3		36.8	3165			254.9	77.4	911	22.5	862.6
DW				8.7		49.6	5880			120.8	23.91	1823	58.4	1921
DX				26.4		35.4	3277			115.4	40.8	2418	38.5	2195
DY				12.7		23.0	4858			92	23.3	1969	35.4	915
DZ				7.8		32.6					84.2	502		66.9
EA				2.9			810			900		650		1000
EB		3.7		2.5	30		680			950	47	800		900
EC							4827.7 ^b^			561.3 ^b^	74.4 ^b^	1058.6 ^b^		1847.3 ^b^

^a^ Median value. ^b^ Mean of the available data in the Paper; nd: not detected.

**Table 3:** Concentration of heavy metals and metalloids in dust samples (values expressed in mg/kg).

Code	As	Cd	Co	Cr	Cu	Hg	Mn	Mo	Ni	Pb	Sb	Zn
AB	5.4–17.7										6.1–232	
AC		1.9		61	2740				69	892		1120
BE										110,000		
BF										22,600		
BO		8.2			1475				130	416		1199
CC		<5		<20	2070	<10		<20		362,000		<10
CD		15.5		64	2140	0.5		4		10,900		
CE		310		86	439	0.5		<2		4600		21100
CF		16.4		21	82	<0.2		<2		1370		506
CN	19.4	4.4	8.87	131.0	1564.1				819.1	89.0		2044.8
CR		42.6		103	1730	3.5		7		88,100		4920
CS		1.4		30	230	<0.2		<2		48		710
CT		97		158	6850	460		12		8615		4440
CU		<5		<20	2670	<10		<20		375,000		21
CV		14.1		78	2800	2.1		7		2360		2200
CW		<0.5		25	414	0.6		<2		100		414
CX		<0.5		25	21	0.5		<2		20		83
CY		200,000		61	1610	48.2		91		13,300		1240
DC		11.4		20	149	<0.2		<2		100		549
DD		2.4			881		509			549	38	1000
EA		3.0			6600					1400		2800
EB	7.4	3.9	33		6300		1800		380	1100		2900

**Table 4:** Concentration of heavy metals and metalloids in sediments (values expressed in mg/kg).

Code	As	Cd	Co	Cr	Cu	Ni	Pb	Zn
AD		0.1		17.6	113	10.1	316	86.8
AP		4.7	14.8	22.1	601	54.5	217	356
AT		0.5		27.3	20.1	12.6	118	175
AX		0		21.2	32.2	20.6	57.7	79.6
AY		0.3		43.5	30.9	20.8	53.1	84.5
AZ		0.9		29.2	528	120	94.3	249
BA		0.2		35.3	66.7	51.5	55.0	133.7
BB		6.3		65.4	2153.9	294.0	394.5	482.8
BG							230	
BH							47.3	
BT	11.9	6.3		316.5	4787.5	153.4	377.3	
DJ	11 ^a^	0.6 ^a^	11 ^a^		400 ^a^	32 ^a^	130 ^a^	200 ^a^

^a^ Median value.

**Table 5:** Concentration of heavy metals and metalloids in air samples (values expressed in ng/m^3^).

Code	Al	As	Cd	Cr	Cu	Mn	Ni	Pb	Sb	Zn
AA	5240		80		570		80	4400	150	3320
AI		10.2 ^a^	7.3 ^a^	1161 ^a^	483 ^a^	60.6 ^a^	10.0 ^a^	444 ^a^		1038 ^a^
AI		6.0 ^b^	7.3 ^b^	1152 ^b^	126 ^b^	25.4 ^b^	7.2 ^b^	392 ^b^		924 ^b^
BL			5.6^b^	6.5 ^b^		22.1 ^b^		153.0 ^b^		
BO			1.1		32.1		4.3	32.9		79.5
CQ			1.5	18	111	59.6		88.9	13	191

^a^ In Total Suspended Particles (TSP); ^b^ In PM2.5.

**Table 6:** Concentration of heavy metals and metalloids in the water (values expressed in mg/L).

Code	Al	As	Cd	Co	Cr	Cu	Ni	Pb	Zn
AU								1.9–24	
BI								0.001–0.002	
CI	3.67	0.04	0.05	0.001	0.60	0.70	0.05	0.04	1.89
CM	61	0.007	0.002	0.001	0.02	0.05	0.03	0.002	1.46

**Table 7:** Concentration of organic pollutants in soil (values expressed in ng/g).

Code	PBDEs	PCDD/Fs	PCBs	Dioxin-Like PCBs
AK	1140 ^a^			
BD	433.8			
BJ		46.1 ^b^		
BU	940 ^a^			
CG			6.5 ^a^	
CH			8.2 ^a^	
DA			148 ^a^	
DB		5 (3.1 × 10^−2^) ^b^		46.1 (3.9 × 10^−2^) ^b^
DF	24			
DG		13 ^b,c^ (PCDDs) 64 ^b,c^ (PCDFs)		
DK	2.2			
DL	1900			
DN		0.7 ^b,c^ (PCDDs) 0.5 ^b,c^ (PCDFs)		
DO		0.8 ^b,c^ (PCDDs) 3.7 ^b,c^ (PCDFs)		
DP		62,000 ^c^ (Total PCDDs) 230,000 ^c^ (Total PCDFs)		42 ^c^
DT		990 ^c^ (Total PCDDs) 2100 ^c^ (Total PCDFs)		1.9 ^c^

^a^ Sum of all the PCBs analyzed; ^b^ In terms of TEQ; ^c^ Median value.

**Table 8:** Concentration of organic pollutants in dust (values expressed in ng/g).

Code	PBDEs	PCDD/Fs	PCB	Dioxin-Like PCBs	PFRs
BN			52 ^a^		33,100 ^a^
BV		0.7 ^b^			
BW			74 ^a^		2180 ^a^
BX			750 ^a^		5560 ^a^
BY			2900 ^a^		6750 ^a^
CA			544 ^a^		7600 ^a^
CB			4.5	1.3	
CD	3000		34,000		
CR			16,000		
CS			25,000		
CT			23,000		
CV	2000		25,000		
CZ			112	58	

^a^ Median value; ^b^ In terms of TEQ.

**Table 9:** Concentration of organic pollutants in sediments (values expressed in pg/g).

Code	PBDEs	PCDD/Fs
DG		1 ^b,c^ (PCDDs) 6.3 ^b,c^ (PCDFs)
DM	243330 ^a^	
DO		1.2 ^b,c^ (PCDDs) 0.04 ^b,c^ (PCDFs)

^a^ Mean of the available data in the paper; ^b^ In terms of TEQ; ^c^ Median value.

**Table 10:** Concentration of organic pollutants in air samples (values expressed in pg/m^3^).

Code	PBDEs (in PM 2.5)	PBDEs (in TSP)	PCDD/Fs (in PM 2.5)	PCDD/Fs (in Particulate and Gas Phase)	PCB
AI	16,822 ^a^			6.5	
AJ	16,600 ^a^	21,500 ^a^			
AN			3.2 ^b^	3.4 ^b^	
BM				24.3 (1.24 ^b^)	
BP				50.2 (0.644 ^b^)	
DR					11,100
DS					4640

^a^ Sum of all the PBDEs analyzed congeners; ^b^ In terms of TEQ.

**Table 11:** Enrichment factors for soils and air samples.

Site	(EF) Cr	(EF) Co	(EF) Ni	(EF) Cu	(EF) Zn	(EF) As	(EF) Cd	(EF) Sb	(EF) Hg	(EF) Pb
SOILS—CI (India)	15.9	13.4	54.4	4198.7	108.5	46.7	221.1	na	24.4	2191
SOILS—CJ(India)	13.7	7.8	29.3	1669.0	126.3	38.7	156.1	na	17.4	nd
SOILS—CK (India)	9.0	6.8	22.6	65.4	32.0	18.6	184.9	na	nd	56.3
SOILS—CL (India)	5.6	3.2	17.6	54.9	24.0	nd	15.7	na	nd	41.0
SOILS—CM (India)	4.3	3.5	18.2	54.3	22.3	nd	10.6	na	nd	39.4
AIR—AA(China)	na	na	50	598.3	1456	489.8	na	11,021	na	7607

nd: not detected; na: not available.

## Supplementary Materials

The following are available online at https://www.mdpi.com/1660-4601/16/9/1595/s1: Bibliography of the studies initially analyzed to compile Table 1.
