# Supplementary material for: WEEE Treatment in Developing Countries: Environmental Pollution and Health Consequences—An Overview
Source: Int J Environ Res Public Health. 2019 May 7;16(9):1595. doi: 10.3390/ijerph16091595 (PMC6539380; doi:10.3390/ijerph16091595)
Supplement: Supplementary file 1 [file ijerph-16-01595-s001.pdf]

International Journal of
Environmental Research and Public Health

MDPI

Supplementary Materials

Bibliography of the studies initially analysed to compile Table 1 (finally, only 50 among the following were included in Table 1)—list in alphabetic order

1. Alabi, O.A.; Bakare, A.A.; Xu, X.; Li, B.; Zhang, Y.; Huo, X. Comparative evaluation of environmental contamination and DNA damage induced by electronic-waste in Nigeria and China. *Sci. Total Environ.* **2012**, *423*, 62–72.
2. Alavi, N.; Shirmardi, M.; Babaei, A.; Takdastan, A.; Bagheri, N. Waste electrical and electronic equipment (WEEE) estimation: A case study of Ahvaz City, Iran. *J. Air Waste Manag. Assoc.* **2015**, *65*, 298–305.
3. Alcántara-Concepción, V.; Gavilán-García, A.; Gavilán-García, I.C. Environmental impacts at the end of life of computers and their management alternatives in México. *J. Clean. Prod.* **2016**, *131*, 615–628.
4. Allenby, B.; Kavazanjian, E.; Kim, J.; Xu, M. Environmental, Social, and Economic Implications of Global Reuse and Recycling of Personal Computers. *Environ. Sci. Technol.* **2008**, *42*, 6446–6454.
5. Asante, K.A.; Agusa, T.; Biney, C.A.; Agyekum, W.A.; Bello, M.; Otsuka, M.; Itai, T.; Takahashi, S.; Tanabe, S. Multi-trace element levels and arsenic speciation in urine of e-waste recycling workers from Agbogbloshie, Accra in Ghana. *Sci. Total Environ.* **2012**, *424*, 63–73.
6. Awasthi, A.K.; Li, J. Management of electrical and electronic waste: A comparative evaluation of China and India. *Renew. Sustain. Energy Rev.* **2017**, *76*, 434–447.
7. Awasthi, A.K.; Wang, M.; Awasthi, M.K.; Wang, Z.; Li, J. Environmental pollution and human body burden from improper recycling of e-waste in China: A short-review. *Environ. Pollut.* **2018**, *243*, 1310–1316.
8. Awasthi, A.K.; Zeng, X.; Li, J. Comparative Examining and Analysis of E-waste Recycling in Typical Developing and Developed Countries. *Procedia Environ. Sci.* **2016**, *35*, 676–680.
9. Awasthi, A.K., Zeng, X., Li, J. Environmental pollution of electronic waste recycling in India: A critical review. *Environ. Pollut.* **2016**, *211*, 259–270.
10. Awasthi, A.K.; Zeng, X.; Li, J. Relationship between e-waste recycling and human health risk in India: A critical review. *Environ. Sci. Pollut. Res.* **2016**, *23*, 11509–11532.
11. Baldé, C.; Forti, V.; Gray, V.; Kuehr, R.; Stegmann, P. *The Global E-Waste Monitor*; United Nations: Bonn, Germany; Geneva, Switzerland; Vienna, Austria, 2017.
12. Bi, X.; Simoneit, B.R.T.; Wang, Z.; Wang, X.; Sheng, G.; Fu, J. The major components of particles emitted during recycling of waste printed circuit boards in a typical e-waste workshop of South China. *Atmos. Environ.* **2010**, *44*, 4440–4445.
13. Bi, X.; Li, Z.; Zhuang, X.; Han, Z.; Yang, W. High levels of antimony in dust from e-waste recycling in southeastern China. *Sci. Total Environ.* **2011**, *409*, 5126–5128.
14. Cai, Z.; Jiang, G. Determination of polybrominated diphenyl ethers in soil from e-waste recycling site. *Talanta* **2006**, *70*, 88–90.
15. Cai, L.M.; Wang, Q.S.; Luo, J.; Chen, L.G.; Zhu, R.L.; Wang, S.; Tang, C.H. Heavy metal contamination and health risk assessment for children near a large Cu-smelter in central China. *Sci. Total Environ.* **2019**, *650*, 725–733.
16. Cao, J.; Xu, X.; Zhang, Y.; Zeng, Z.; Hylkema, M.N.; Huo, X. Increased memory T cell populations in Pb-exposed children from an e-waste-recycling area. *Sci. Total Environ.* **2018**, *616–617*, 988–995.
17. Capraz, O.; Polat, O.; Gungor, A. Planning of waste electrical and electronic equipment (WEEE) recycling facilities: MILP modelling and case study investigation. *Flex. Serv. Manuf. J.* **2015**, *27*, 479–508.
18. Chakraborty, P.; Prithiviraj, B.; Selvaraj, S.; Kumar, B. Polychlorinated biphenyls in settled dust from informal electronic waste recycling workshops and nearby highways in urban centers and suburban industrial roadsides of Chennai city, India: Levels, congener profiles and exposure assessment. *Sci. Total Environ.* **2016**, *573*, 1413–1421.
19. Chakraborty, P.; Selvaraj, S.; Nakamura, M.; Prithiviraj, B.; Cincinelli, A.; Bang, J.J. PCBs and PCDD/Fs in soil from informal e-waste recycling sites and open dumpsites in India: Levels, congener profiles and health risk assessment. *Sci. Total Environ.* **2018**, *621*, 930–938.

20. Chan, J.K.Y.; Wong, M.H. A review of environmental fate, body burdens, and human health risk assessment of PCDD/Fs at two typical electronic waste recycling sites in China. *Sci. Total Environ.* **2013**, *463–464*, 1111–1123.
21. Chi, X.; Wang, M.Y.L.; Reuter, M.A. E-waste collection channels and household recycling behaviors in Taizhou of China. *J. Clean. Prod.* **2014**, *80*, 87–95.
22. Cong, X.; Xu, X.; Xu, L.; Li, M.; Xu, C.; Qin, Q.; Huo, X. Elevated biomarkers of sympatho-adrenomedullary activity linked to e-waste air pollutant exposure in preschool children. *Environ. Int.* **2018**, *115*, 117–126.
23. Cucchiella, F.; D'Adamo, I.; Lenny Koh, S.C.; Rosa, P. Recycling of WEEEs: An economic assessment of present and future e-waste streams. *Renew. Sustain. Energy Rev.* **2015**, *51*, 263–272.
24. Dai, Y.; Huo, X.; Zhang, Y.; Yang, T.; Li, M.; Xu, X. Elevated lead levels and changes in blood morphology and erythrocyte CR1 in preschool children from an e-waste area. *Sci. Total Environ.* **2017**, *592*, 51–59.
25. Damrongsiri, S.; Vassanadumrongdee, S.; Tanwattana, P. Heavy metal contamination characteristic of soil in WEEE (waste electrical and electronic equipment) dismantling community: A case study of Bangkok, Thailand. *Environ. Sci. Pollut. Res.* **2016**, *23*, 17026–17034.
26. Danciu, A.M.; Greenley, M.; Cobuz, A.P. An overview of global e-waste, its effects on developing countries and possible solutions. *Rev. Appl. Socioecon. Res.* **2018**, *15*, 20–28.
27. Deng, W.J.; Louie, P.K.K.; Liu, W.K.; Bi, X.H.; Fu, J.M.; Wong, M.H. Atmospheric levels and cytotoxicity of PAHs and heavy metals in TSP and PM2.5 at an electronic waste recycling site in southeast China. *Atmos. Environ.* **2006**, *40*, 6945–6955.
28. Deng, W.J.; Zheng, J.S.; Bi, X.H.; Fu, J.M.; Wong, M.H. Distribution of PBDEs in air particles from an electronic waste recycling site compared with Guangzhou and Hong Kong, South China. *Environ. Int.* **2007**, *33*, 1063–1069.
29. Duan, H.; Li, J.; Liu, Y.; Yamazaki, N.; Jiang, W. Characterizing the emission of chlorinated/brominated dibenzo-p-dioxins and furans from low-temperature thermal processing of waste printed circuit board. *Environ. Pollut.* **2012**, *161*, 185–191.
30. Eguchi, A.; Nomiyama, K.; Devanathan, G.; Subramanian, A.; Bulbule, K.A.; Parthasarathy, P.; Takahashi, S.; Tanabe, S. Different profiles of anthropogenic and naturally produced organohalogen compounds in serum from residents living near a coastal area and e-waste recycling workers in India. *Environ. Int.* **2012**, *47*, 8–16.
31. Fu, J.; Zhou, Q.; Liu, J.; Liu, W.; Wang, T.; Zhang, Q.; Jiang, G. High levels of heavy metals in rice (Oryza sativa L.) from a typical E-waste recycling area in southeast China and its potential risk to human health. *Chemosphere* **2008**, *71*, 1269–1275.
32. Fujimori, T.; Itai, T.; Goto, A.; Asante, K.A.; Otsuka, M.; Takahashi, S.; Tanabe, S. Interplay of metals and bromine with dioxin-related compounds concentrated in e-waste open burning soil from Agbogbloshie in Accra, Ghana. *Environ. Pollut.* **2016**, *209*, 155–163.
33. Fujimori, T.; Takigami, H.; Agusa, T.; Eguchi, A.; Bekki, K.; Yoshida, A.; Terazono, A.; Ballesteros, F.C. Impact of metals in surface matrices from formal and informal electronic-waste recycling around Metro Manila, the Philippines, and intra-Asian comparison. *J. Hazard. Mater.* **2012**, *221–222*, 139–146.
34. Grant, K.; Goldizen, F.C.; Sly, P.D.; Brune, M.N.; Neira, M.; van den Berg, M.; Norman, R.E. Health consequences of exposure to e-waste: A systematic review. *Lancet Glob. Heal.* **2013**, *1*, e350–e361.
35. Gullett, B.K.; Linak, W.P.; Touati, A.; Wasson, S.J.; Gatica, S.; King, C.J. Characterization of air emissions and residual ash from open burning of electronic wastes during simulated rudimentary recycling operations. *J. Mater. Cycles Waste Manag.* **2007**, *9*, 69–79.
36. Ha, N.N.; Agusa, T.; Ramu, K.; Tu, N.P.C.; Murata, S.; Bulbule, K.A.; Parthasaraty, P.; Takahashi, S.; Subramanian, A.; Tanabe, S. Contamination by trace elements at e-waste recycling sites in Bangalore, India. *Chemosphere* **2009**, *76*, 9–15.
37. Ha, N.N.; Agusa, T.; Ramu, K.; Tu, N.P.C.; Murata, S.; Bulbule, K.A.; Parthasaraty, P.; Takahashi, S.; Subramanian, A.; Tanabe, S. Contamination by trace elements at e-waste recycling sites in Bangalore, India. *Chemosphere* **2009**, *76*, 9–15.
38. Hai, H.T.; Hung, H.V.; Quang, N.D. An overview of electronic waste recycling in Vietnam. *J. Mater. Cycles Waste Manag.* **2017**, *19*, 536–544.
39. Herat, S.; Agamuthu, P. E-waste: A problem or an opportunity? Review of issues, challenges and solutions in Asian countries. *Waste Manag. Res.* **2012**, *30*, 1113–1129.

40. Hoeltl, A.; Brandtweiner, R.; Müller, R. Approach to solving the E-waste problem—Case study Ghana. *Int. J. Sustain. Dev. Plan.* **2017**, *12*, 1050–1060.
41. Hogarh, J.N.; Seike, N.; Kobara, Y.; Carboo, D.; Fobil, J.N.; Masunaga, S. Source characterization and risk of exposure to atmospheric polychlorinated biphenyls (PCBs) in Ghana. *Environ. Sci. Pollut. Res.* **2018**, *25*, 16316–16324.
42. Hong, J.; Shi, W.; Wang, Y.; Chen, W.; Li, X. Life cycle assessment of electronic waste treatment. *Waste Manag.* **2015**, *38*, 357–365.
43. Ilankoon, I.M.S.K.; Ghorbani, Y.; Chong, M.N.; Herath, G.; Moyo, T.; Petersen, J. E-waste in the international context—A review of trade flows, regulations, hazards, waste management strategies and technologies for value recovery. *Waste Manag.* **2018**, *82*, 258–275.
44. Imran, M.; Haydar, S.; Kim, J.; Awan, M.R.; Bhatti, A.A. E-waste flows, resource recovery and improvement of legal framework in Pakistan. *Resour. Conserv. Recycl.* **2017**, *125*, 131–138.
45. Iqbal, M.; Breivik, K.; Syed, J.H.; Malik, R.N.; Li, J.; Zhang, G.; Jones, K.C. Emerging issue of e-waste in Pakistan: A review of status, research needs and data gaps. *Environ. Pollut.* **2015**, *207*, 308–318.
46. Isimekhai, K.A.; Garelick, H.; Watt, J.; Purchase, D. Heavy metals distribution and risk assessment in soil from an informal E-waste recycling site in Lagos State, Nigeria. *Environ. Sci. Pollut. Res.* **2017**, *24*, 17206–17219.
47. Itai, T.; Otsuka, M.; Asante, K.A.; Muto, M.; Opoku-Ankomah, Y.; Ansa-Asare, O.D.; Tanabe, S. Variation and distribution of metals and metalloids in soil/ash mixtures from Agbogbloshie e-waste recycling site in Accra, Ghana. *Sci. Total Environ.* **2014**, *470–471*, 707–716.
48. Jibiri, N.N.; Isinkaye, M.O.; Momoh, H.A. Assessment of radiation exposure levels at Alaba e-waste dumpsite in comparison with municipal waste dumpsites in southwest Nigeria. *J. Radiat. Res. Appl. Sci.* **2014**, *7*, 536–541.
49. Jun-hui, Z.; Hang, M. Eco-toxicity and metal contamination of paddy soil in an e-wastes recycling area. *J. Hazard. Mater.* **2009**, *165*, 744–750.
50. Kiddee, P.; Naidu, R.; Wong, M.H. Electronic waste management approaches: An overview. *Waste Manag.* **2013**, *33*, 1237–1250.
51. Kumar, A.; Holuszko, M.; Espinosa, D.C.R. E-waste: An overview on generation, collection, legislation and recycling practices. *Resour. Conserv. Recycl.* **2017**, *122*, 32–42.
52. Labunska, I.; Abdallah, M.A.E.; Eulaers, I.; Covaci, A.; Tao, F.; Wang, M.; Santillo, D.; Johnston, P.; Harrad, S. Human dietary intake of organohalogen contaminants at e-waste recycling sites in Eastern China. *Environ. Int.* **2015**, *74*, 209–220.
53. Laha, S. Informality in E-Waste Processing: An Analysis of the Indian Experience. *Compet. Chang.* **2014**, *18*, 309–326.
54. Leung, A.; Cai, Z.W.; Wong, M.H. Environmental contamination from electronic waste recycling at Guiyu, southeast China. *J. Mater. Cycles Waste Manag.* **2006**, *8*, 21–33.
55. Leung, A.O.W.; Duzgoren-Aydin, N.S.; Cheung, K.C.; Wong, M.H. Heavy metals concentrations of surface dust from e-waste recycling and its human health implications in southeast China. *Environ. Sci. Technol.* **2008**, *42*, 2674–2680.
56. Li, J.; Duan, H.; Shi, P. Heavy metal contamination of surface soil in electronic waste dismantling area: Site investigation and source-apportionment analysis. *Waste Manag. Res.* **2011**, *29*, 727–738.
57. Li, J.; Lopez, N., B.N.; Liu, L.; Zhao, N.; Yu, K.; Zheng, L. Regional or global WEEE recycling. Where to go? *Waste Manag.* **2013**, *33*, 923–934.
58. Li, J.; Yang, J.; Liu, L. Development potential of e-waste recycling industry in China. *Waste Manag. Res.* **2015**, *33*, 533–542.
59. Li, H.; Yu, L.; Sheng, G.; Fu, J.; Peng, P. Severe PCDD/F and PBDD/F pollution in air around an electronic waste dismantling area in China. *Environ. Sci. Technol.* **2007**, *41*, 5641–5646.
60. Liu, H.; Zhou, Q.; Wang, Y.; Zhang, Q.; Cai, Z.; Jiang, G. E-waste recycling induced polybrominated diphenyl ethers, polychlorinated biphenyls, polychlorinated dibenzo-p-dioxins and dibenzo-furans pollution in the ambient environment. *Environ. Int.* **2008**, *34*, 67–72.
61. Li, T.Y.; Zhou, J.F.; Wu, C.C.; Bao, L.J.; Shi, L.; Zeng, E.Y. Characteristics of Polybrominated Diphenyl Ethers Released from Thermal Treatment and Open Burning of E-Waste. *Environ. Sci. Technol.* **2018**, *52*, 4650–4657.

62. Leung, A.; Cai, Z.W.; Wong, M.H. Environmental contamination from electronic waste recycling at Guiyu, southeast China. *J. Mater. Cycles Waste Manag.* **2006**, *8*, 21–33.
63. Luo, C.; Liu, C.; Wang, Y.; Liu, X.; Li, F.; Zhang, G.; Li, X. Heavy metal contamination in soils and vegetables near an e-waste processing site, south China. *J. Hazard. Mater.* **2011**, *186*, 481–490.
64. Ma, J.; Kannan, K.; Cheng, J.; Horii, Y.; Wu, Q.; Wang, W. Concentrations, profiles, and estimated human exposures for polychlorinated dibenzo-p-dioxins and dibenzofurans from electronic waste recycling facilities and a chemical industrial complex in Eastern China. *Environ. Sci. Technol.* **2008**, *42*, 8252–8259.
65. Man, M.; Naidu, R.; Wong, M.H. Persistent toxic substances released from uncontrolled e-waste recycling and actions for the future. *Sci. Total Environ.* **2013**, *463–464*, 1133–1137.
66. Matsukami, H.; Tue, N.M.; Suzuki, G.; Someya, M.; Tuyen, L.H.; Viet, P.H.; Takahashi, S.; Tanabe, S.; Takigami, H. Flame retardant emission from e-waste recycling operation in northern Vietnam: Environmental occurrence of emerging organophosphorus esters used as alternatives for PBDEs. *Sci. Total Environ.* **2015**, *514*, 492–499.
67. Milovantseva, N.; Fitzpatrick, C. Barriers to electronics reuse of transboundary e-waste shipment regulations: An evaluation based on industry experiences. *Resour. Conserv. Recycl.* **2015**, *102*, 170–177.
68. Mueller, S.R.; Wäger, P.A.; Widmer, R.; Williams, I.D. A geological reconnaissance of electrical and electronic waste as a source for rare earth metals. *Waste Manag.* **2015**, *45*, 226–234.
69. Nie, X.; Fan, C.; Wang, Z.; Su, T.; Liu, X.; An, T. Toxic assessment of the leachates of paddy soils and river sediments from e-waste dismantling sites to microalga, Pseudokirchneriella subcapitata. *Ecotoxicol. Environ. Saf.* **2015**, *111*, 168–176.
70. Nnorom, I.C.; Ohakwe, J.; Osibanjo, O. Survey of willingness of residents to participate in electronic waste recycling in Nigeria—A case study of mobile phone recycling. *J. Clean. Prod.* **2009**, *17*, 1629–1637.
71. Nnorom, I.C.; Osibanjo, O. Electronic waste (e-waste): Material flows and management practices in Nigeria. *Waste Manag.* **2008**, *28*, 1472–1479.
72. Nnorom, I.C.; Osibanjo, O. Sound management of brominated flame retarded (BFR) plastics from electronic wastes: State of the art and options in Nigeria. *Resour. Conserv. Recycl.* **2008**, *52*, 1362–1372.
73. Oguri, T.; Suzuki, G.; Matsukami, H.; Uchida, N.; Tue, N.M.; Tuyen, L.H.; Viet, P.H.; Takahashi, S.; Tanabe, S.; Takigami, H. Exposure assessment of heavy metals in an e-waste processing area in northern Vietnam. *Sci. Total Environ.* **2018**, *621*, 1115–1123.
74. Olafisoye, O.B.; Adefioye, T.; Osibote, O.A. Heavy metals contamination of water, soil, and plants around an electronic waste dumpsite. *Polish J. Environ. Stud.* **2013**, *22*, 1431–1439.
75. Ongondo, F.O.; Williams, I.D.; Cherrett, T.J. How are WEEE doing? A global review of the management of electrical and electronic wastes. *Waste Manag.* **2011**, *31*, 714–730.
76. Orlins, S.; Guan, D. China's toxic informal e-waste recycling: Local approaches to a global environmental problem. *J. Clean. Prod.* **2016**, *114*, 71–80.
77. Perkins, D.N.; Brune Drisse, M.N.; Nxele, T.; Sly, P.D. E-waste: A global hazard. *Ann. Glob. Heal.* **2014**, *80*, 286–295.
78. Pradhan, J.K.; Kumar, S. Informal e-waste recycling: Environmental risk assessment of heavy metal contamination in Mandoli industrial area, Delhi, India. *Environ. Sci. Pollut. Res.* **2014**, *21*, 7913–7928.
79. Quan, S.X.; Yan, B.; Yang, F.; Li, N.; Xiao, X.M.; Fu, J.M. Spatial distribution of heavy metal contamination in soils near a primitive e-waste recycling site. *Environ. Sci. Pollut. Res.* **2015**, *22*, 1290–1298.
80. Rochat, D.; Hagelüken, C.; Keller, M.; Widmer, R. Optimal recycling for printed wiring boards (PWBs) in India. *Conf. Recover. Mater. Energy Resour. Effic.* **2007**, 12.
81. Scruggs, C.E.; Nimpuno, N.; Moore, R.B.B. Improving information flow on chemicals in electronic products and E-waste to minimize negative consequences for health and the environment. *Resour. Conserv. Recycl.* **2016**, *113*, 149–164.
82. Sepúlveda, A., Schluep, M., Renaud, F.G., Streicher, M., Kuehr, R., Hagelüken, C., Gerecke, A.C. A review of the environmental fate and effects of hazardous substances released from electrical and electronic equipments during recycling: Examples from China and India. *Environ. Impact Assess. Rev.* **2010**, *30*, 28–41.
83. Sinha-Khetriwal, D.; Kraeuchi, P.; Schwaninger, M. A comparison of electronic waste recycling in Switzerland and in India. *Environ. Impact Assess. Rev.* **2005**, *25*, 492–504.
84. Singh, M.; Thind, P.S.; John, S. Health risk assessment of the workers exposed to the heavy metals in e-waste recycling sites of Chandigarh and Ludhiana, Punjab, India. *Chemosphere* **2018**, *203*, 426–433.

85. Song, Q.; Li, J. A review on human health consequences of metals exposure to e-waste in China. *Environ. Pollut.* **2015**, *196*, 450–461.
86. Song, Q.; Li, J. Environmental effects of heavy metals derived from the e-waste recycling activities in China: A systematic review. *Waste Manag.* **2014**, *34*, 2587–2594.
87. Song, Q.; Zeng, X.; Li, J.; Duan, H.; Yuan, W. Environmental risk assessment of CRT and PCB workshops in a mobile e-waste recycling plant. *Environ. Sci. Pollut. Res.* **2015**, *22*, 12366–12373.
88. Steuer, B.; Ramusch, R.; Part, F.; Salhofer, S. Analysis of the value chain and network structure of informal waste recycling in Beijing, China. *Resour. Conserv. Recycl.* **2017**, *117*, 137–150.
89. Sthiannopkao, S.; Wong, M.H. Handling e-waste in developed and developing countries: Initiatives, practices, and consequences. *Sci. Total Environ.* **2013**, *463–464*, 1147–1153.
90. Streicher-Porte, M.; Widmer, R.; Jain, A.; Bader, H.P.; Scheidegger, R.; Kytzia, S. Key drivers of the e-waste recycling system: Assessing and modelling e-waste processing in the informal sector in Delhi. *Environ. Impact Assess. Rev.* **2005**, *25*, 472–491.
91. Suzuki, G.; Someya, M.; Matsukami, H.; Tue, N.M.; Uchida, N.; Tuyen, L.H.; Viet, P.H.; Takahashi, S.; Tanabe, S.; Brouwer, A.; et al. Comprehensive evaluation of dioxins and dioxin-like compounds in surface soils and river sediments from e-waste-processing sites in a village in northern Vietnam: Heading towards the environmentally sound management of e-waste. *Emerg. Contam.* **2016**, *2*, 98–108.
92. Tang, X.; Shen, C.; Chen, L.; Xiao, X.; Wu, J.; Khan, M.I.; Dou, C.; Chen, Y. Inorganic and organic pollution in agricultural soil from an emerging e-waste recycling town in Taizhou area, China. *J. Soils Sediments* **2010**, *10*, 895–906.
93. Tang, X.; Shen, C.; Shi, D.; Cheema, S.A.; Khan, M.I.; Zhang, C.; Chen, Y. Heavy metal and persistent organic compound contamination in soil from Wenling: An emerging e-waste recycling city in Taizhou area, China. *J. Hazard. Mater.* **2010**, *173*, 653–660.
94. Tang, X.; Shen, C.; Chen, L.; Xiao, X.; Wu, J.; Khan, M.I.; Dou, C.; Chen, Y. Inorganic and organic pollution in agricultural soil from an emerging e-waste recycling town in Taizhou area, China. *J. Soils Sediments* **2010**, *10*, 895–906.
95. Tansel, B. From electronic consumer products to e-wastes: Global outlook, waste quantities, recycling challenges. *Environ. Int.* **2017**, *98*, 35–45.
96. Tsydenova, O.; Bengtsson, M. Chemical hazards associated with treatment of waste electrical and electronic equipment. *Waste Manag.* **2011**, *31*, 45–58.
97. Tue, N.M.; Goto, A.; Takahashi, S.; Itai, T.; Asante, K.A.; Kunisue, T.; Tanabe, S. Release of chlorinated, brominated and mixed halogenated dioxin-related compounds to soils from open burning of e-waste in Agbogbloshie (Accra, Ghana). *J. Hazard. Mater.* **2016**, *302*, 151–157.
98. Tue, N.M.; Goto, A.; Takahashi, S.; Itai, T.; Asante, K.A.; Nomiyama, K.; Tanabe, S.; Kunisue, T. Soil contamination by halogenated polycyclic aromatic hydrocarbons from open burning of e-waste in Agbogbloshie (Accra, Ghana). *J. Mater. Cycles Waste Manag.* **2017**, *19*, 1324–1332.
99. Tran, C.D.; Salhofer, S.P. Analysis of recycling structures for e-waste in Vietnam. *J. Mater. Cycles Waste Manag.* **2018**, *20*, 110–126.
100. Uchida, N.; Matsukami, H.; Someya, M.; Tue, N.M.; Tuyen, L.H.; Viet, P.H.; Takahashi, S.; Tanabe, S.; Suzuki, G. Hazardous metals emissions from e-waste-processing sites in a village in northern Vietnam. *Emerg. Contam.* **2018**, *4*, 11–21.
101. Umair, S.; Björklund, A.; Petersen, E.E. Social impact assessment of informal recycling of electronic ICT waste in Pakistan using UNEP SETAC guidelines. *Resour. Conserv. Recycl.* **2015**, *95*, 46–57.
102. Wang, F.; Huisman, J.; Meskers, C.E.M.; Schluep, M.; Stevels, A.; Hagelüken, C. The Best-of-2-Worlds philosophy: Developing local dismantling and global infrastructure network for sustainable e-waste treatment in emerging economies. *Waste Manag.* **2012**, *32*, 2134–2146.
103. Wang, J.; Liu, L.; Wang, J.; Pan, B.; Fu, X.; Zhang, G.; Zhang, L.; Lin, K. Distribution of metals and brominated flame retardants (BFRs) in sediments, soils and plants from an informal e-waste dismantling site, South China. *Environ. Sci. Pollut. Res.* **2015**, *22*, 1020–1033.
104. Wang, F.; Zhao, Y.; Zhang, T.; Duan, C.; Wang, L. Mineralogical analysis of dust collected from typical recycling line of waste printed circuit boards. *Waste Manag.* **2015**, *43*, 434–441.
105. Wen, S.; Gong, Y.; Li, J.G.; Shi, T.M.; Zhao, Y.F.; Wu, Y.N. Particle-bound PCDD/Fs in the Atmosphere of an Electronic Waste Dismantling Area in China. *Biomed. Environ. Sci.* **2011**, *24*, 102–111.

106. Wen, S.; Yang, F.X.; Gong, Y.; Zhang, X.L.; Hui, Y.; Li, J.G.; Liu, A.I.L.; Wu, Y.N.; Lu, W.Q.; Xu, Y. Elevated levels of urinary 8-hydroxy-2′-deoxyguanosine in male electrical and electronic equipment dismantling workers exposed to high concentrations of polychlorinated dibenzo-p-dioxins and dibenzofurans, polybrominated diphenyl ethers, and polychlorinat. *Environ. Sci. Technol.* **2008**, *42*, 4202–4207.
107. Wibowo, S.; Deng, H. Multi-criteria group decision making for evaluating the performance of e-waste recycling programs under uncertainty. *Waste Manag.* **2015**, *40*, 127–135.
108. Wittsiepe, J.; Fobil, J.N.; Till, H.; Burchard, G.D.; Wilhelm, M.; Feldt, T. Levels of polychlorinated dibenzo-p-dioxins, dibenzofurans (PCDD/Fs) and biphenyls (PCBs) in blood of informal e-waste recycling workers from Agbogbloshie, Ghana, and controls. *Environ. Int.* **2015**, *79*, 65–73.
109. Wong, C.S.C.; Duzgoren-Aydin, N.S.; Aydin, A.; Wong, M.H. Evidence of excessive releases of metals from primitive e-waste processing in Guiyu, China. *Environ. Pollut.* **2007**, *148*, 62–72.
110. Wong, C.S.C.; Wu, S.C.; Duzgoren-Aydin, N.S.; Aydin, A.; Wong, M.H. Trace metal contamination of sediments in an e-waste processing village in China. *Environ. Pollut.* **2007**, *145*, 434–442.
111. Wong, M.H.; Wu, S.C.; Deng, W.J.; Yu, X.Z.; Luo, Q.; Leung, A.O.W.; Wong, C.S.C.; Luksemburg, W.J.; Wong, A.S. Export of toxic chemicals—A review of the case of uncontrolled electronic-waste recycling. *Environ. Pollut.* **2007**, *149*, 131–140.
112. Yang, X.; Sun, L.; Xiang, J.; Hu, S.; Su, S. Pyrolysis and dehalogenation of plastics from waste electrical and electronic equipment (WEEE): A review. *Waste Manag.* **2013**, *33*, 462–473.
113. Yoshida, A.; Terazono, A.; Ballesteros, F.C.; Nguyen, D.Q.; Sukandar, S.; Kojima, M.; Sakata, S. E-waste recycling processes in Indonesia, the Philippines, and Vietnam: A case study of cathode ray tube TVs and monitors. *Resour. Conserv. Recycl.* **2016**, *106*, 48–58.
114. Yu, Y.; Zhu, X.; Li, L.; Lin, B.; Xiang, M.; Zhang, X.; Chen, X.; Yu, Z.; Wang, Z.; Wan, Y. Health implication of heavy metals exposure via multiple pathways for residents living near a former e-waste recycling area in China: A comparative study. *Ecotoxicol. Environ. Saf.* **2019**, *169*, 178–184.
115. Yuan, J.; Chen, L.; Chen, D.; Guo, H.; Bi, X.; Ju, Y.; Jiang, P.; Shi, J.; Yu, Z.; Yang, J.; et al. Elevated Serum Polybrominated Diphenyl Ethers and Thyroid-Stimulating Hormone Associated with Lymphocytic Micronuclei in Chinese Workers from an E-Waste Dismantling Site. *Environ. Sci. Technol.* **2008**, *42*, 2195–2200.
116. Zeng, X.; Duan, H.; Wang, F.; Li, J. Examining environmental management of e-waste: China’s experience and lessons. *Renew. Sustain. Energy Rev.* **2017**, *72*, 1076–1082.
117. Zeng, X.; Song, Q.; Li, J.; Yuan, W.; Duan, H.; Liu, L. Solving e-waste problem using an integrated mobile recycling plant. *J. Clean. Prod.* **2015**, *90*, 55–59.
118. Zeng, X.; Xu, X.; Zheng, X.; Reponen, T.; Chen, A.; Huo, X. Heavy metals in PM2.5and in blood, and children’s respiratory symptoms and asthma from an e-waste recycling area. *Environ. Pollut.* **2016**, *210*, 346–353.
119. Zhao, W.; Ding, L.; Gu, X.; Luo, J.; Liu, Y.; Guo, L.; Shi, Y.; Huang, T.; Cheng, S. Levels and ecological risk assessment of metals in soils from a typical e-waste recycling region in southeast China. *Ecotoxicology* **2015**, *24*, 1947–1960.
120. Zhang, M.; Feng, G.; Yin, W.; Xie, B.; Ren, M.; Xu, Z.; Zhang, S.; Cai, Z. Airborne PCDD/Fs in two e-waste recycling regions after stricter environmental regulations. *J. Environ. Sci. (China)* **2017**, *62*, 3–10.
121. Zhang, Y.; Huo, X.; Cao, J.; Yang, T.; Xu, L.; Xu, X. Elevated lead levels and adverse effects on natural killer cells in children from an electronic waste recycling area. *Environ. Pollut.* **2016**, *213*, 143–150.
122. Zhang, T.; Ruan, J.; Zhang, B.; Lu, S.; Gao, C.; Huang, L.; Bai, X.; Xie, L.; Gui, M.; Qiu, R.L. Heavy metals in human urine, foods and drinking water from an e-waste dismantling area: Identification of exposure sources and metal-induced health risk. *Ecotoxicol. Environ. Saf.* **2019**, *169*, 707–713.
123. Zheng, X.; Xu, F.; Chen, K.; Zeng, Y.; Luo, X.; Chen, S.; Mai, B.; Covaci, A. Flame retardants and organochlorines in indoor dust from several e-waste recycling sites in South China: Composition variations and implications for human exposure. *Environ. Int.* **2015**, *78*, 1–7.
124. Zhao, W.; Ding, L.; Gu, X.; Luo, J.; Liu, Y.; Guo, L.; Shi, Y.; Huang, T.; Cheng, S. Levels and ecological risk assessment of metals in soils from a typical e-waste recycling region in southeast China. *Ecotoxicology* **2015**, *24*, 1947–1960.
125. Zhang, M.; Feng, G.; Yin, W.; Xie, B.; Ren, M.; Xu, Z.; Zhang, S.; Cai, Z. Airborne PCDD/Fs in two e-waste recycling regions after stricter environmental regulations. *J. Environ. Sci. (China)* **2017**, *62*, 3–10.

126. Zhang, Y.; Huo, X.; Cao, J.; Yang, T.; Xu, L.; Xu, X. Elevated lead levels and adverse effects on natural killer cells in children from an electronic waste recycling area. *Environ. Pollut.* **2016**, *213*, 143–150.
127. Zhu, Z.; Han, Z.; Bi, X.; Yang, W. The relationship between magnetic parameters and heavy metal contents of indoor dust in e-waste recycling impacted area, Southeast China. *Sci. Total Environ.* **2012**, *433*, 302–308.
128. Zhang, T.; Ruan, J.; Zhang, B.; Lu, S.; Gao, C.; Huang, L.; Bai, X.; Xie, L.; Gui, M.; Qiu, R.L. Heavy metals in human urine, foods and drinking water from an e-waste dismantling area: Identification of exposure sources and metal-induced health risk. *Ecotoxicol. Environ. Saf.* **2019**, *169*, 707–713.

© 2019 by the authors. Licensee MDPI, Basel, Switzerland. This article is an open access article distributed under the terms and conditions of the Creative Commons Attribution (CC BY) license (http://creativecommons.org/licenses/by/4.0/).
